# The adverse impact of coronary artery disease on left ventricle systolic and diastolic function in patients with type 2 diabetes mellitus: a 3.0T CMR study

**DOI:** 10.1186/s12933-022-01467-y

**Published:** 2022-02-22

**Authors:** Jin Wang, Yuan Li, Ying-Kun Guo, Shan Huang, Rui Shi, Wei-Feng Yan, Wen-Lei Qian, Guang-Xi He, Zhi-Gang Yang

**Affiliations:** 1 Department of Radiology, West China Hospital, Sichuan University, 37# Guo Xue Xiang, Chengdu, 610041 Sichuan China; 2 Department of Radiology, Key Laboratory of Obstetric & Gynecologic and Pediatric Diseases and Birth Defects of Ministry of Education, West China Second University Hospital, Sichuan University, 20# Section 3, Renmin South Road, Chengdu, 610041 Sichuan China

**Keywords:** Type 2 diabetes mellitus, Coronary artery disease, Left ventricular, Strain, Magnetic resonance imaging

## Abstract

**Background:**

Coronary artery disease (CAD) confers considerable morbidity and mortality in diabetes. However, the role of CAD in additive effect of left ventricular (LV) function has rarely been explored in type 2 diabetes mellitus (T2DM) patients. This study aimed to investigate how CAD affect LV systolic and diastolic function in T2DM patients.

**Materials and methods:**

A total of 282 T2DM patients {104 patients with CAD [T2DM (CAD +)] and 178 without [T2DM (CAD −)]} and 83 sex- and age- matched healthy controls underwent cardiac magnetic resonance scanning. LV structure, function, global strains [including systolic peak strain (PS), peak systolic (PSSR) and diastolic strain rate (PDSR) in radial, circumferential and longitudinal directions] and late gadolinium enhancement (LGE) parameters were measured. T2DM (CAD +) patients were divided into two subgroups based on the median of Gensini score (60) which was calculated to assess the severity of CAD. Multivariable linear regression analyses were constructed to investigate the determinants of reduced LV function.

**Results:**

Compared with normal controls, T2DM (CAD −) patients exhibited increased LV end-diastolic and end-systolic volume index and decreased LV global strains, while T2DM(CAD +) patients showed more marked increase and decrease than T2DM(CAD-) and healthy controls, except for longitudinal PDSR (PDSR-L) (all *P* < 0.017). All of LV global strains demonstrated a progressive decrease from normal controls, through Gensini score ≤ 60, to Gensini score > 60 group, except for PDSR-L (all *P* < 0.017). CAD was an independent predictor of reduced LV global circumferential PS (GCPS, β = 0.22, *p* < 0.001), PSSR (PSSR-C, β = 0.17, *p* = 0.005), PDSR (PDSR-C, β = 0.22, *p* < 0.001), global radial PS (GRPS, β = 0.19, *p* = 0.001), and global longitudinal PS (GLPS, β = 0.18, *p* = 0.003) in T2DM. The Gensini score was associated with decreased GCPS, PSSR-C, PDSR-C, GRPS, and GLPS in T2DM (CAD +) (all *p* < 0.05).

**Conclusion:**

CAD has an additive deleterious effect on LV systolic and diastolic function in T2DM patients. Among T2DM (CAD +) patients, the Gensini score is associated with reduced LV contractile and diastolic function.

*Trial registration* Retrospectively registered

## Background

All forms of cardiovascular disease are common complications of diabetes mellitus that confers considerable morbidity and mortality [1, 2]. Mounting evidence suggest that type 2 diabetes mellitus (T2DM) can directly affect the myocardium, resulting in microvascular dysfunction, myocardial fibrosis, and left ventricular (LV) hypertrophy [3, 4]. Furthermore, it is well documented that T2DM associated with an exaggerated risk of macrovascular complication, such as coronary artery disease (CAD) [5]. People with T2DM have an almost two- to four- fold higher risk of CAD compared with those without [6]. Under the presence of ischemia, CAD may aggravate detrimental LV remodeling and dysfunction in patients with T2DM, which leads to advanced heart failure and poor clinical outcomes [7]. Therefore, a better understanding of the underlying influences of CAD on T2DM regarding cardiac function may be of paramount importance to improve our management efforts and reduce the incidence of long-term adverse events.

Cardiac magnetic resonance (CMR) tissue tracking is a novel method of measuring LV strain and strain rates, which has been applied to evaluate the LV systolic or diastolic function in diverse populations and shown high sensitivity and reproducibility [8–10]. To the best of our knowledge, several previous studies have focused on the additive effect of hypertension or valvular regurgitation on the LV function in T2DM patients [11, 12], the role of CAD on which has rarely been explored in those patients. [13] Accordingly, this study aimed to investigate how CAD affect LV systolic and diastolic function in T2DM patients and further to explore the independent determinants of decreased LV function.

## Materials and methods

### Study population

Between January 2015 and July 2021, 488 patients with T2DM undergoing CMR at our institution were retrospectively enrolled in this study. Among them, those patients with chest pain and suspected CAD were selected to undergo invasive coronary angiography (ICA) examination. T2DM was defined according to the current American Diabetes Association guidelines [14]. Obstructive CAD was defined when the luminal diameter stenosis was estimated as ≥ 50% in at least one major epicardial coronary artery [15]. Smoking was defined as current or previous smoking of at least one cigarette per day for at least 1 year [16]. Exclusion criteria were: (1) patients with prior percutaneous or surgical revascularization procedures, (2) congenital or valvular heart disease, (3) primary cardiomyopathy, (4) severe renal failure (estimated glomerular filtration rate < 30 mL/min), (5) the maximum percentage of luminal stenosis < 50% in any segment of major coronary arteries with patients who were diagnosed with CAD, (6) contradictions to CMR imaging, (7) and poor image quality. Finally, 282 T2DM patients (mean age, 59.00 ± 11.52 years; 183 male) who met above criteria were included in this study and these patients were categorized into two groups: T2DM with CAD [T2DM (CAD +), n = 104] and T2DM without CAD [T2DM (CAD −), n = 178]. In addition, 83 age- and sex-matched healthy individuals (mean age, 56.45 ± 10.05 years;  55 male) who underwent CMR examination were recruited to serve as the normal control group with no history of cardiovascular or systematic disease or diabetes mellitus. This study protocol was approved by the Biomedical Research Ethics Committee of our hospital and conducted in accordance with the ethical guidelines of the Declaration of Helsinki. Written informed consent was obtained from all subjects.

### Invasive coronary angiography

Selective ICA was performed through the radial or femoral artery approach by interventional cardiologists who were blinded to the clinical data using a standard Judkins technique. T2DM patients with CAD were classified into one-, two-, or three-vessel disease according to the number of diseased coronary arteries. The luminal diameter narrowing of left main coronary artery ≥ 50% was considered as three-vessel disease [17]. According to the method described in the literature [18], the Gensini score was calculated to assess the severity of CAD by two independent experienced cardiologists who were blinded to the clinical and procedural data.

### CMR protocol

All participants underwent CMR examination using a 3.0T whole-body scanner (MAGNETOM Skyra; Siemens Medical Solutions, Erlangen, Germany) in the supine position. Continuous data acquisition was performed during the end-inspiratory breath-holding period using the manufacturer’s standard ECG-triggering device which monitored dynamic changes in each subject’s ECG findings. A balanced steady-state free precession (bSSFP) sequence (TR/TE 3.4/1.2 ms, field of view 340 × 280.5 mm, flip angle 38°, slice thickness 8 mm, and matrix size 192 × 162) was performed to obtain a stack of contiguous cine images covering the whole LV from the base to the apex in the short-axis slices, as well as the four-, three-, and two-chamber in the long-axis views. Subsequently, a dose of 0.2 mL/kg body weight gadobenate dimeglumine (MultiHance 0.5 mmol/mL; Bracco, Milan, Italy) was intravenously injected at a flow rate of 2.5–3.0 mL/s, then a 20-mL saline flush immediately was injected at a rate of 3.0 mL/s. Segmented − turbo − FLASH − phase − sensitive inversion recovery (PSIR) sequence (TR/TE 598 /3.31 ms, field of view 340 × 255 mm, flip angle 40°, slice thickness 8 mm, and matrix size 256 × 162) was used to acquire LGE images after 10–15 min of contrast administration.

### CMR image analysis

CMR images of all participants were evaluated offline using commercial software (cvi^42^, Circle Cardiovascular Imaging Inc., Calgary, Alberta, Canada) by two radiologists, each of whom had more than 5 years of CMR experience and were blinded to clinical data.

The epicardial and endocardial borders of the LV myocardium on a stack of short-axis cine images were manually outlined at the end-systolic and end-diastolic phases using the afore-mentioned software. Then, cardiac geometry and function parameters including LV mass (LVM) at end-diastole, LV end-diastolic volume (LVEDV), LV end-systolic volume (LVESV), LV stroke volume (LVSV), and LV ejection fraction (LVEF) were automatically computed. According to the Mosteller formula, LVM, LVEDV, and LVESV indexed for body surface area (BSA) were calculated and represented as LVMI, LVEDVI, and LVESVI, retrospectively [19]. LV papillary muscles and moderate bands were included in the measures of LV volume and excluded from the LVM.

A set of LV short-axis cine images in combination with two- and four- chamber long-axis images were loaded into the tissue tracking module to evaluate three-dimensional (3D) strain of LV myocardial. The LV global strain parameters including systolic peak strain (PS), peak systolic strain rate (PSSR), and peak diastolic strain rate (PDSR) in the three directions (radial, circumferential, and longitudinal) were obtained automatically using the above software. By convention, the radial strains were positive during systolic phase with radial thickening. While the longitudinal and circumferential strains were negative during systole with longitudinal and circumferential shortening respectively.

In addition, the presence or absence of LGE was analyzed by those above radiologists. A threshold- defined as 5 standard deviations (SDs) above the signal of the remote normal myocardial region-was used to measure the extent of LGE [20].

### Reproducibility of LV global strains

To analyze intra-observer variability, the same observer with more than 5 years of CMR experience compared the LV global strains in 40 randomly selected participants including 30 T2DM patients and 10 healthy individuals with 2-month interval. Inter-observer variability was also obtained from the same population and was assessed by comparing the independent measurements by a second observer who is independent and double-blinded with more than 5 years of CMR experience.

### Statistical analysis

All statistical analyses were performed using IBM SPSS Statistics for Windows version 24.0 (IBM Corporation, Armonk, NY, USA) and GraphPad Prism version 7.0a (GraphPad Software, San Diego, California, USA). The Shapiro–Wilk test was performed to evaluate data for normality and Levene’s test for homogeneity of continuous variables. The continuous normally distributed data were expressed as mean ± SD and non-normally distributed variables were expressed as the median (interquartile range, 25–75%). Discrete data were presented as number (percentage) and compared with Fisher’s exact test. Parameters among T2DM (CAD −), T2DM (CAD +) and normal controls groups were compared by one-way analysis of variance (One-way ANOVA) followed by Bonferroni’s post hoc-test or the Kruskal–Wallis rank test, where appropriate. The same methods were also applied to compare the parameters among T2DM (CAD +) patients with different Gensini score groups and normal controls group. Univariable linear regression analyses were constructed to identify the correlations of LV global strains with multiple clinical risk factors and other imaging parameters. Variables with an absence of collinearity and *p* values < 0.1 in the univariable analyses were selected in stepwise multivariable linear regression models to evaluate the independent effect of CAD on LV function in T2DM patients or identify the determinants of reduced LV function among T2DM (CAD +) patients. Inter- and intra-observer agreements were assessed using the intra-class correlation coefficient (ICC). A two-tailed *p* value of < 0.05 was considered to indicate statistical significance.

## Results

### Baseline characteristics

The baseline characteristics of the study cohort are presented in Table 1. Compared to T2DM(CAD-) patients and normal controls, T2DM(CAD +) patients were older, more often men and smokers, as well as had larger body mass index (BMI) and BSA, and had higher levels of fasting blood glucose, HbA1c, and N-terminal pro-brain natriuretic peptide (all *p* < 0.017). T2DM (CAD +) patients had longer disease duration than those T2DM (CAD −) [4.5 (0.63, 10) vs. 2 (0, 7.5) years, *p* < 0.05). The levels of blood pressure, triglycerides and renal function were similar in both subgroups of T2DM patients with and without CAD (all *p* > 0.05).

**Table 1:** Baseline characteristics of the study population

	Normal controls	T2DM	
	(n = 83)	T2DM (CAD** −**) (n = 178)	T2DM (CAD** +**) (n = 104)
Age (years)	56.45 ± 10.05	57.33 ± 12.12	61.86 ± 9.83^*§^
Male, n (%)	55(66.3%)	101(56.7%)	82 (78.8%) ^§^
BMI (kg/m^2^)	21.24 ± 3.44	22.58 ± 9.58	25.47 ± 4.34^*§^
BSA (m^2^)	1.58 ± 0.15	1.52 ± 0.57	1.76 ± 0.19^*§^
Smoking, n (%)	8(9.6%)	62(34.8%) ^*^	52(51.5%) ^*§^
Systolic blood pressure (mmHg)	111.66 ± 10.54	134.21 ± 21.12^*^	131.59 ± 22.01^*^
Diastolic blood pressure (mmHg)	74.17 ± 6.11	82.05 ± 14.12^*^	80.43 ± 14.43^*^
Heart rate (beats/min)	71.93 ± 5.67	83.81 ± 15.32^*^	80.28 ± 13.84^*^
Fasting blood glucose (mmol/L)	4.9 (4.5, 5.1)	7.2 (5.7, 9.3) ^*^	8.9 (6.7, 13.2) ^*§^
HbA1c, (%)	5.1 (4.9, 5.4)	6.8 (6.4, 7.4) ^*^	7.9 (7.3, 8.6) ^*§^
TG (mmol/L)	1.0 (0.7, 1.3)	1.5 (1.0, 2.1) ^*^	1.4 (1.0, 2.2) ^*^
TC (mmol/L)	3.8 (3.1, 4.5)	4.0 (3.2, 4.7)	3.8 (3.1, 4.6)
HDL (mmol/L)	1.2 (1.1, 1.5)	1.1 (0.9, 1.4) ^*^	1.0 (0.9, 1.2) ^*^
LDL (mmol/L)	2.1 (1.6, 2.8)	2.1 (1.6, 2.7)	2.0 (1.5, 2.7)
eGFR (mL/min/1.73 m^2^)	91.6 (81, 99.9)	80.0 (62.2, 96.2) ^*^	79.1 (67.2, 92.3) ^*^
NT-proBNP, (pg/mL)	51 (29,60)	394 (131,1494) ^*^	509 (208, 2020) ^*§^
Diabetes duration (years)	–	2 (0, 7.5)	4.5 (0.63, 10) ^§^
Gensini score	–	–	60 (39, 97)
Number of coronary arteries affected			
One/Two/Three-vessel	–	–	34/30/40
Location of coronary artery occlusion			
(LAD/LCX/RCA)	–	–	95/52/66

All values are presented as mean ± SD or n (%) or median (Q1-Q3)

*T2DM* type 2 diabetes mellitus, *CAD* coronary artery disease, *BSA* body surface area, *BMI* body mass index, *HbA1c* glycated haemoglobin, *TG* triglycerides, *TC* total cholesterol, *HDL* high-density lipoprotein, *LDL* low-density lipoprotein, *eGFR* estimated glomerular filtration rate, *NT-proBNP* N-terminal pro-brain natriuretic peptide, *LAD* left descending artery, *LCX* left circumflex artery, *RCA* right coronary artery

*P < 0.017 vs. normal controls; ^§^P < 0.017 vs. T2DM (CAD −) group

Among 104 T2DM (CAD +) patients, the median (IQR) Gensini score was 60 (39, 97) and the number (%) of patients for one-, two-, and three-vessel disease were 34 (32.69%), 30 (28.85%), and 40 (38.46%) respectively. Regarding the location of CAD, 95 (91.35%) patients had left ascending artery stenosis, 52 (50%) patients had left circumflex artery stenosis, and 66 (63.46%) patients had right coronary stenosis. No significant differences in management of antidiabetic drugs were observed between the T2DM (CAD −) and T2DM(CAD +) groups (all *p* > 0.05) (Table 2).

**Table 2:** Treatment in patients with T2DM

	T2DM (CAD** −**) (n = 178)	T2DM (CAD +) (n = 104)	*P* value
Diabetic treatment			
Biguanides, n (%)	59 (33.1%)	43 (41.4%)	0.167
Sulfonylureas, n (%)	31 (17.4%)	18 (17.3%)	0.982
a-Glucosidase inhibitor, n (%)	48 (27%)	31 (29.8%)	0.608
GLP-1/DPP-4 inhibitor, n (%)	9 (5%)	5 (4.8%)	0.926
Insulin, n (%)	46 (25.8%)	38 (36.5%)	0.058
Diet controlled	60 (33.7%)	22 (21.2%)	0.025
CAD treatment			
Drugs			
Aspirin, n (%)	–	83 (79.8%)	–
Clopidogrel, n (%)	–	73 (70.2%)	–
Warfarin, n (%)	–	3 (2.9%)	–
Statins, n (%)	–	89 (85.6%)	–
Beta blockers, n (%)	–	44 (42.3%)	–
ACEI, ARB, n (%)	–	40 (38.5%)	–
Nitrate, n (%)	–	8 (7.7%)	–
Trimetazidine hydrochloride, n (%)	–	14 (13.5%)	–
Calcium antagonists, n (%)	–	26 (25%)	–
PCI	–	64 (61.5%)	–
CABG	–	3 (2.9%)	–

All values are presented as n (%). *GLP-1/DPP-4 inhibitor* glucagon-like peptide-1/dipeptidyl peptidase 4 inhibitors, *PCI* percutaneous coronary intervention, *CABG* coronary artery bypass grafting, *ACEI* angiotensin converting enzyme inhibitor

### Comparison of CMR-derived indices among T2DM patients with and without CAD, and normal controls

**Fig. 1:**
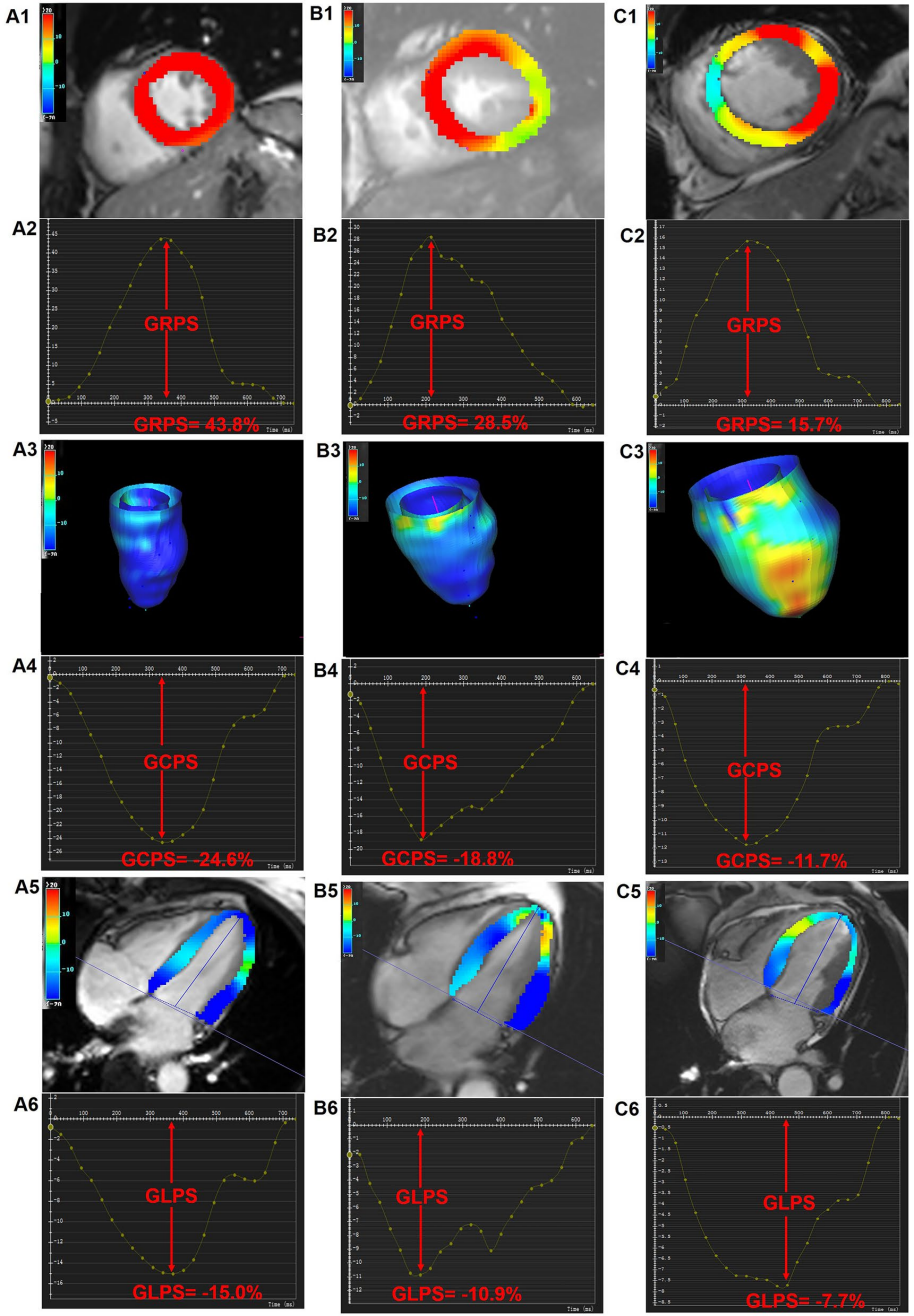
Representative CMR pseudo-color images at the end-systole and global PS curves in a normal control, T2DM (CAD −) patient, and T2DM (CAD +) patient. **A1**–**C1** pseudo-color images of LV radial PS in short-axis; **A3**–**C3** three-dimensional pseudo-color maps of LV circumferential PS; **A5**, **B5**, **C5** pseudo-color images of LV longitudinal PS in four-chamber long-axis; **A2**–**C2** LV global PS curve in radial direction; **A4**, **B4**, **C4** LV global PS curve in circumferential direction; **A6**, **B6**, **C6** LV global PS curve in longitudinal direction. *CMR* cardiac magnetic resonance, *PS* peak strain, *LV* left ventricle, *T2DM* type 2 diabetes mellitus, *CAD* coronary artery disease

The CMR findings are shown in Table 3 and Fig. 1. In contrast to the normal controls, both T2DM patients with and without CAD exhibited increased LVMI and enlarged LVEDV and LVESV (all *p* < 0.017). LVEF, as preserved in those without CAD, showed a significant progressive decrease from normal controls, through T2DM (CAD −), to T2DM (CAD +) patients [63.1 (58, 67.8) vs. 55.1 (40.5, 63.5) vs. 37.2 (27, 52.4), *p* < 0.017]. T2DM (CAD +) patients showed a higher presence [104 (100%) vs. 56 (31.5%), *p* < 0.05] and extent of LGE [22.6 (12.9, 46.3) % vs. 7.7 (4.1, 16.8) %, *p* < 0.05) than those T2DM (CAD −).

**Table 3:** Comparison of CMR-derived parameters among normal controls, T2DM patients with and without CAD

	Normal controls		T2DM	
	(n = 83)	T2DM (CAD** −**) (n = 178)		T2DM (CAD** +**) (n = 104)
LV geometry and function				
LVEDVI (mL/m^2^)	79.0 (67.0, 87.7)	89.3 (74.6, 117.5)^*^		106.5 (79.0, 141.4)^*§^
LVESVI (mL/m^2^)	28.0 (23.9, 33.1)	37.5 (26.9, 72.9)^*^		59.2 (36.1, 107.8)^*§^
LVSVI (mL/m^2^)	49.8 (42.3, 54.4)	46.7 (35.5, 55.5)		40.1 (32.0, 48.6)^*§^
LVEF (%)	63.1 (58.0, 67.8)	55.1 (40.5, 63.5)^*^		37.2 (27.0, 52.4)^*§^
LVMI (g/m^2^)	42.0 (35.4, 47.5)	51.5 (43.1, 68.4)^*^		59.3 (50.1, 66.6)^*^
PS (%)				
GRPS	35.3 (30.4, 38.8)	24.2 (13.4, 33.6) ^*^		18.1 (9.5, 23.9) ^*§^
GCPS	− 20.8 (− 22.2, − 19.1)	− 16.9 (− 20.7, − 10.6) ^*^		− 12.2 (− 16.3, − 7.5) ^*§^
GLPS	− 14.4 (− 16.7, − 12.2)	− 10.0 (− 12.5, − 5.9) ^*^		− 7.8 (− 10.0, − 5.2) ^*§^
PSSR (1/s)				
Radial	2.0 (1.7, 2.4)	1.4 (0.7, 1.9)^*^		1.0 (0.6, 1.5)^*§^
Circumferential	− 1.0 (− 1.2, − 0.9)	− 0.9 (− 1.1, − 0.6)^*^		− 0.7 (− 0.8, − 0.5)^*§^
Longitudinal	− 0.8 (− 0.9, − 0.6)	− 0.7 (− 0.8, − 0.4)^*^		− 0.5 (− 0.7, − 0.4)^*§^
PDSR (1/s)				
Radial	** − **2.5 (− 3.1, − 2.0)	** − **1.5 (− 2.1, − 0.8)^*^		− 1.1 (− 1.5, − 0.6) ^*§^
Circumferential	1.3 (1.1, 1.5)	0.9 (0.6, 1.1)^*^		0.7 (0.5, 0.9)^*§^
Longitudinal	0.9 (0.7, 1.1)	0.6 (0.5, 0.8)^*^		0.6 (0.4, 0.7) ^*^
LGE				
LGE, n (%)	–	56 (31.5%)		104 (100%) ^§^
LGE rel (%)	–	7.7 (4.1, 16.8) %		22.6 (12.9, 46.3)% ^§^

All values are presented as n (%) or median (Q1-Q3). “**−**” indicates the direction of strains. *LV* left ventricular, *EF* ejection fraction, *EDV* end-diastolic volume, *ESV* end-systolic volume, *SV* stroke-volume, *I* indexed to BSA, *PS* peak strain, *GRPS* global radial peak strain, *GCPS* global circumferential peak strain, *GLPS* global longitudinal peak strain, *PSSR* peak systolic strain rate, *PDSR* peak diastolic strain rate, *LGE* late gadolinium enhancement

*P < 0.017 vs. normal group; ^§^P < 0.017 vs. T2DM (CAD-) group

Regarding the LV global myocardial strains, T2DM patients even absence of CAD had evidence of decreased systolic and diastolic function, with a significant more impaired PS, PSSR, and PDSR in all of three directions than normal controls (all *p* < 0.017). Compared with the T2DM (CAD −) patients and normal controls, the decrease in all of LV global strains (PS, PSSR, and PDSR in the radial, circumferential, and longitudinal directions) among those T2DM (CAD +) was more marked, except for longitudinal PDSR (PDSR-L) (all *p* < 0.017).

### Comparison of LV global strains among T2DM (CAD +) patients with different Gensini score and normal controls

**Fig. 2:**
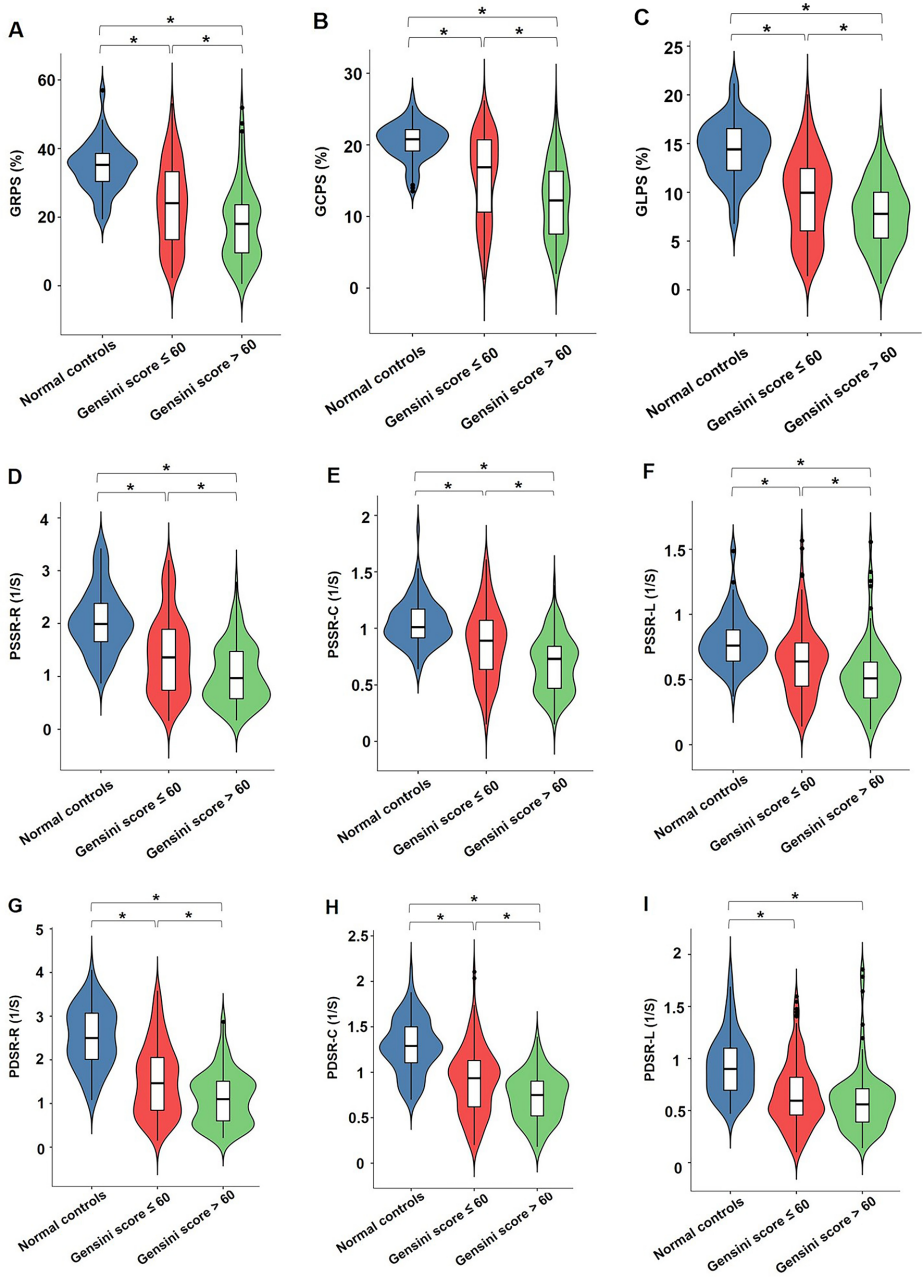
Comparison of LV global strains among T2DM (CAD +) patients with different Gensini score and normal controls. The absolute values of LV global strains were used to avoid the influence of directional sign. *PS* peak strain, *GRPS* global radial peak strain, *GCPS* global circumferential peak strain, *GLPS* global longitudinal peak strain, *PSSR* peak systolic strain rate, *PDSR* peak diastolic strain rate, *R* radial, *C* circumferential, *L* longitudinal, *T2DM* type 2 diabetes mellitus, *CAD* coronary artery disease. *P < 0.017

According to the median value (60) of the Gensini score, T2DM (CAD +) patients were divided into two subgroups: T2DM (CAD +) patients with Gensini score ≤ 60 (n = 51) and those with Gensini score > 60 (n = 53). The CMR-derived LV global strain parameters for the observed groups are demonstrated in Fig. 2. All of the LV global strains declined progressively from normal controls, through T2DM (CAD +) patients with Gensini score ≤ 60, to those with Gensini score > 60 (all *p* < 0.017), whereas the PDSR-L showed no significant difference between subgroups of T2DM (CAD +) patients with different Gensini score (*p* > 0.05).

### Independent effect of CAD on LV systolic and diastolic function in T2DM patients

Multivariable linear regression analyses were performed to evaluate the independent effect of CAD on LV systolic and diastolic function in T2DM patients (Table 4). After controlling for sex, age, BSA, BMI, systolic and diastolic blood pressure, heart rate, and diabetes duration, CAD was independently associated with LV global circumferential PS (GCPS, β = 0.22, *p* < 0.001), PSSR (PSSR-C, β = 0.17, *p* = 0.005), PDSR (PDSR-C, β = 0.22, *p* < 0.001), global radial PS (GRPS, β = 0.19, *p* = 0.001) and global longitudinal PS (GLPS, β = 0.18, *p* = 0.003).

**Table 4:** Effect of CAD on the LV systolic and diastolic function in T2DM patients

	GRPS (%)		GLPS (%)		GCPS (%)		PSSR-C (1/s)		PDSR-C (1/s)	
	Univariable	Multivariable	Univariable	Multivariable	Univariable	Multivariable	Univariable	Multivariable	Univariable	Multivariable
	r	β	r	β	r	β	r	β	r	β
CAD	0.23^§^	0.19^*^	0.21^§^	0.18^*^	0.28^§^	0.22^*^	0.27^§^	0.17^*^	0.27^§^	0.22^*^
Sex	** − **0.30^§^	** − **0.25^*^	** − **0.29^§^	** − **0.26^*^	** − **0.33^§^	** − **0.24^*^	** − **0.19^§^	N/A	** − **0.26^§^	−0.22^*^
Age	** − **0.02	N/A	** − **0.02	N/A	** − **0.02	N/A	** − **0.10^§^	N/A	** − **0.09	N/A
BMI	** − **0.001	N/A	** − **0.04	N/A	** − **0.03	N/A	0.01	N/A	− 0.06	N/A
BSA	** − **0.10	N/A	** − **0.12^§^	N/A	** − **0.14^§^	N/A	** − **0.09	N/A	** − **0.17^§^	N/A
SBP	0.17^§^	N/A	0.10^§^	N/A	0.20^§^	0.17^*^	0.17^§^	0.13^*^	0.11^§^	N/A
DBP	0.01	N/A	** − **0.02	N/A	0.03	N/A	0.05	N/A	0.05	N/A
Heart rate	** − **0.14^§^	** − **0.13^*^	** − **0.16^§^	** − **0.17^*^	** − **0.16^§^	** − **0.18^*^	** − **0.10	N/A	** − **0.03	N/A
Smoking	** − **0.18^§^	N/A	** − **0.20^§^	N/A	** − **0.24^§^	N/A	** − **0.16^§^	N/A	** − **0.24^§^	N/A
Diabetes duration	** − **0.02	N/A	** − **0.04	N/A	** − **0.01	N/A	** − **0.06	N/A	** − **0.07	N/A

β is adjusted regression coefficient

Factors with *P* < 0.1 in the univariable analyses were included in the stepwise multiple liner regression model

^§^*P* < 0.1

*P < 0.05

Abbreviations as in Tables 1, 3

### Determinants of impaired LV systolic and diastolic function in T2DM (CAD +) patients

**Fig. 3:**
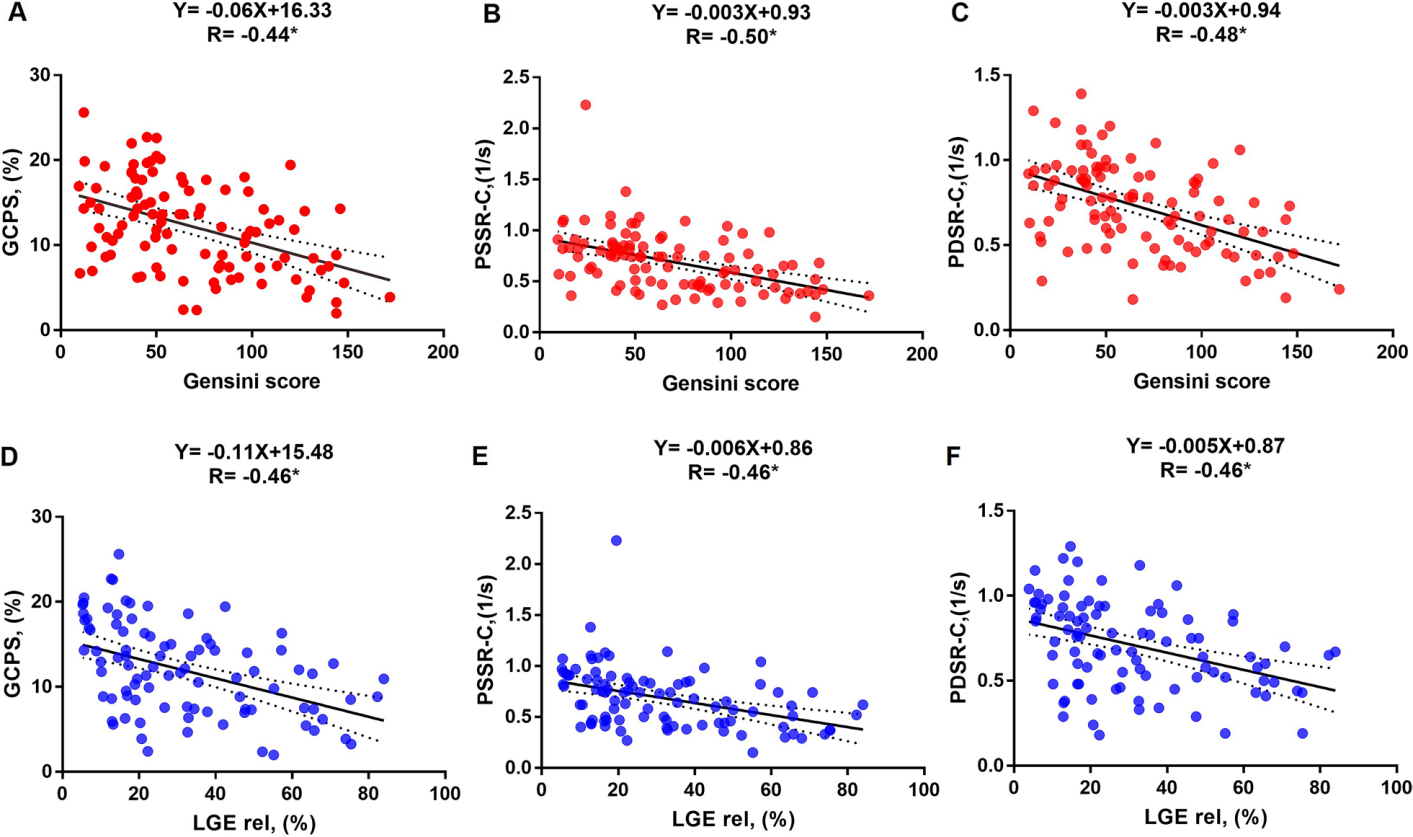
The associations of LV global circumferential strains with Gensini score and LGE in T2DM (CAD +) patients. The absolute values of LV circumferential global peak strains were used to avoid the influence of directional sign. *GCPS* global circumferential peak strain, *PSSR-C* the global circumferential peak systolic strain rate, *PDSR-C* the global circumferential peak diastolic strain rate, *T2DM* type 2 diabetes mellitus, *CAD* coronary artery disease. *P < 0.001

The univariable analyses in T2DM (CAD +) patients exhibited that the Gensini score was negatively associated with GCPS (r = − 0.44, *p* < 0.001), PSSR-C (r = − 0.50, *p* < 0.001), PDSR-C (r = − 0.48, *p* < 0.001) (Fig. 3 A-C), GRPS (r = − 0.43, *p* < 0.001), and GLPS (r = − 0.43, *p* < 0.001). In addition, there were negative correlations between the extent of LGE and GCPS (r = − 0.46, *p* < 0.001), PSSR-C (r = − 0.46, *p* < 0.001), and PDSR-C (r = − 0.46, *p* < 0.001) (Fig. 3D–F), GRPS (r = − 0.44, *p* < 0.001), and GLPS (r = − 0.37, *p* < 0.001). Increasing NT-proBNP level was also significantly associated with worsening GRPS (r = − 0.49, *p* < 0.001), GCPS (r = − 0.52, *p* < 0.001), and GLPS (r = − 0.55, *p* < 0.001).

After adjusting for confounding factors, the Gensini score remained the independent determinant of impaired GCPS (β = − 0.34, *p* < 0.001), PSSR-C (β = − 0.35, *p* < 0.001), PDSR-C (β = − 0.34, *p* < 0.001), GRPS (β = − 0.29, *p* = 0.001) and GLPS (β = − 0.35, *p* < 0.001) (Table 5).

**Table 5:** Determinants of impaired LV systolic and diastolic function in T2DM(CAD +) patients

	GRPS (%)		GLPS (%)		GCPS (%)		PSSR-C (1/s)		PDSR-C (1/s)	
	Univariable	Multivariable	Univariable	Multivariable	Univariable	Multivariable	Univariable	Multivariable	Univariable	Multivariable
	r	β	r	β	r	β	r	β	r	β
Sex	**− **0.31^§^	**− **0.23^*^	**− **0.22^§^	N/A	**− **0.25^§^	N/A	**− **0.31^§^	N/A	**− **0.24^§^	N/A
Age	0.18^§^	N/A	0.15	N/A	0.21^§^	N/A	0.14	N/A	0.10	N/A
Smoking	**− **0.21^§^	N/A	**− **0.20^§^	N/A	**− **0.25^§^	**− **0.21^*^	**− **0.25^§^	N/A	**− **0.27^§^	** − **0.23^*^
BMI	0.10	N/A	0.06	N/A	0.04	N/A	0.10	N/A	0.10	N/A
BSA	**− **0.04	N/A	**− **0.10	N/A	**− **0.11	N/A	0.12	N/A	**− **0.10	N/A
Heart rate	**− **0.29^§^	N/A	**− **0.34^§^	**− **0.24^*^	**− **0.35^§^	**− **0.27^*^	**− **0.20^§^	N/A	**− **0.16	N/A
SBP	0.32^§^	N/A	0.28	N/A	0.33^§^	N/A	0.32^§^	N/A	0.26^§^	N/A
DBP	0.10	N/A	0.07	N/A	0.11	N/A	0.12	N/A	0.13	N/A
Nt-proBNP^#^	**− **0.49^§^	**− **0.27^*^	**− **0.55^§^	**− **0.29^*^	**− **0.52^§^	**− **0.21^*^	**− **0.49^§^	**− **0.21^*^	**− **0.48^§^	**− **0.20^*^
Diabetes duration	0.14	N/A	0.15	N/A	0.15	N/A	0.03	N/A	0.01	N/A
HbA1c	**− **0.11	N/A	**− **0.14	N/A	**− **0.18^§^	N/A	**− **0.13	N/A	**− **0.19	N/A
Gensini score	**− **0.43^§^	**− **0.29^*^	**− **0.43^§^	**− **0.35^*^	**− **0.44^§^	**− **0.34^*^	**− **0.50^§^	**− **0.35^*^	**− **0.48^§^	**− **0.34^*^
LGE rel	**− **0.44^§^	**− **0.28^*^	**− **0.37^§^	**− **0.23^*^	**− **0.46^§^	**− **0.27^*^	**− **0.46^§^	**− **0.29^*^	**− **0.46^§^	**− **0.26^*^

β is adjusted regression coefficient

^#^NT-proBNP is log-transformed before being included in the regression model

Factors with *P* < 0.1 in the univariable analyses were included in the stepwise multiple liner regression model

^§^*P* < 0.1

*P < 0.05

Abbreviations as in Tables 1, 3

### Inter-observer and intra-observer variability of LV strain parameters

As shown in Table 6, there were significantly high inter-observer and intra-observer agreements of LV global myocardial strains measured with CMR tissue tracking. The coefficient of variation of inter-observer variability for LV global PS, PSSR, and PDSR were 0.905–0.960, 0.893–0.909, and 0.895–0.944 respectively. The coefficient of variation of intra-observer variability for PS, PSSR, and PDSR were 0.900–0.969, 0.901–0.939, and 0.908–0.951 respectively.

**Table 6:** Inter-and intra-observer variabilities of LV myocardial strain

	Inter-observer (n = 40)		Intra-observer (n = 40)	
	ICC	95% CI	ICC	95% CI
PS (%)				
GRPS	0.932	0.859–0.966	0.969	0.943–0.984
GCPS	0.960	0.926–0.979	0.951	0.900–0.975
GLPS	0.905	0.829–0.949	0.900	0.820–0.946
PSSR (1/s)				
Radial	0.893	0.803–0.943	0.925	0.863–0.959
Circumferential	0.909	0.836–0.951	0.939	0.888–0.967
Longitudinal	0.896	0.812–0.944	0.901	0.822–0.947
PDSR (1/s)				
Radial	0.944	0.882–0.972	0.951	0.909–0.974
Circumferential	0.902	0.823–0.947	0.932	0.875–0.963
Longitudinal	0.895	0.804–0.944	0.908	0.834–0.950

*ICC* intraclass correlation coefficient, *CI* confidence interval, *PS* peak strain, *GRPS* global radial peak strain, *GCPS* global circumferential peak strain, *GLPS* global longitudinal peak strain, *PSSR* peak systolic strain rate, *PDSR* peak diastolic strain rate

## Discussion

The present study highlights evidence supporting the viewpoint that CAD has a deleterious effect on LV function in T2DM patients. The key findings were as follows: (1) even in absence of CAD, T2DM patients displayed an adverse LV remodeling and reduced LV systolic and diastolic function, (2) when coexisting CAD, T2DM patients demonstrated a significantly more severe impairment of systolic and diastolic function, with a more marked reduction in nearly all of the LV strains than those without CAD and normal controls, (3) after adjusting for confounding factors, CAD was an independent predictor of reduced LV systolic and diastolic function in T2DM patients. (4) among T2DM (CAD +) patients, LV strains declined progressively along with the increase of Gensini score, in addition, the Gensini score was the independent determinant of decreased LV systolic and diastolic function.

Previous trials have suggested that adverse LV remodeling and dysfunction can occur even in absence of CAD in diabetes, as demonstrated by increased LVMI, with diastolic dysfunction, either alone or combined with depressed systolic function [21, 22]. The present study showed similar changes in cardiac structure and exhibited reduced both systolic and diastolic function in T2DM (CAD −) patients, despite with preserved LVEF. Besides, the presence of LGE indicating myocardial fibrosis was also observed in these patients. Therefore, detection of early changes in the LV myocardium allows for early intervention and instigating preventative strategies in T2DM patients.

Available data regarding the CAD-related influence on LV function in T2DM patients remain limited [13]. In this study, our data exhibited that T2DM (CAD +) patients had a more significantly marked decreased in nearly all of LV global strains, larger LVESVI, and lower LVEF than those T2DM (CAD −) and normal individuals, which suggest that CAD exacerbate the impairment of systolic and diastolic function. The precise underlying mechanisms of the adverse impact of CAD on LV systolic and diastolic function are not fully understood but are likely to be multifactorial. The potential mechanistic explanations for this finding are as follows: on the one hand, hyperglycemia, metabolic-related disorders, and insulin resistance induce states of pro-thrombotic, pro-inflammatory, and pro-oxidant in the context of DM, which contribute to calcium handing altered, excitation–contraction coupling impairment, myocardium interstitial fibrosis, and microvasculature abnormalities [23, 24]. Some of above-mentioned changes in molecular signal pathways also exist in a milieu of CAD [25, 26]. When T2DM comorbid with CAD, we speculate that those superimposed factors in myocardium might be amplified, thereby promoting the aggravation of myocardial contractility and relaxation impairment.

On the other hand, the association between myocardium ischemia and obstructive coronary stenosis was well established [27]. In addition, the presence and extent of LGE in our T2DM (CAD +) patients was higher than that in T2DM (CAD −) patients. Those above suggested the potential pathophysiological mechanisms of myocardium ischemia and increased international fibrosis [28] which result in more severe impairment of systolic and diastolic function in T2DM(CAD +) patients than those in T2DM (CAD −) patients. Future researches will be encouraged to explore the exact molecular mechanisms of these two stimuli (CAD and DM) interaction regarding affecting myocardial contractile and diastolic dysfunction.

Gensini score is one of the angiographic scoring systems which is widely used for quantifying the severity of CAD [29]. Accumulating studies indicated that CAD was associated with poor clinical outcomes (such as myocardial infarction, death) in T2DM patients [30, 31]. Importantly, our results add additional information by showing that LV systolic and diastolic function (nearly all of the LV global strains) decreased progressively along with the aggravation of CAD (increase of Gensini score) among T2DM(CAD +) patients. In addition, the Gensini score was the independent determinant of reduced LV contractile and diastolic function. These results emphasized the significance of CAD management in patients with T2DM. Currently, coronary revascularization either with percutaneous coronary intervention (PCI) or with coronary artery bypass graft (CABG) is acceptable option in individuals with DM and more extensive CAD [32, 33]. A recent large study based on the ISCHEMIA Trials [34] demonstrated that patients with chronic coronary disease and diabetes did not derive incremental benefit from routine coronary revascularization in contrast to the initial medical therapy alone. It is highly stimulating the exploration of future targeted medicine therapy in order to improve management and prognosis of high-risk patients with DM and CAD.

Anderson et al. [35] reported that the presence of myocardial scar detected by CMR was significantly correlated with the LV circumferential deformation in patients with DM, which was in concordance with previous study by Jiang et al. [21] In the present study, we found there was also a significant association of LGE with LV longitudinal, circumferential, and radial deformation in T2DM (CAD +) patients, which was similar to those prior observations and extended those findings to patients with both DM and CAD.

### Limitations

Our results should be interpreted in the context of several limitations. First, this study was a single-center and retrospective study, and the likelihood for selection bias cannot be disregarded. Besides, insulin levels, insulin resistance (HOMA-IR) and inflammatory markers (such as: CRP and IL-6) were not complete for all patients. Their role as possible predictors of CAD in T2DM patients will be explored in the future prospective study. Second, although multivariable adjustments were conducted to obtain significant confounders, we cannot exclude the existence of unmeasured covariates. Third, although not all of our T2DM (CAD −) patients underwent coronary angiography, including invasive coronary angiography or coronary computed tomography angiography, CAD was deemed to be unlikely according to the comprehensive assessment of the patients by clinical history, electrocardiography, laboratory results and echocardiography, subsequently supported by the CMR examinations [21]. Finally, it is a cross-sectional analysis of T2DM patients with CAD and the evolution of cardiac remodeling and function is not known over time as the CAD progression. Future longitudinal studies will be needed to address this question.

## Conclusions

In summary, CAD had an additive deleterious effect on LV systolic and diastolic function in patients with T2DM. The Gensini score quantifying the severity of CAD was associated with reduced contractile and diastolic function among T2DM (CAD +) patients. Future studies should be encouraged to explore the underlying mechanisms by which the coexistence of these two stimuli (CAD and DM) affect the myocardium and how these effects could be prevented.

## Data Availability

The datasets used and analyzed during the current study are available from the corresponding author on reasonable request.

## Abbreviations

CAD: Coronary artery disease
T2DM: Type 2 diabetes mellitus
LV: Left ventricular
PS: Peak strain
PSSR: Peak systolic strain rate
PDSR: Peak diastolic strain rate
LGE: Late gadolinium enhancement
PDSR-L: The longitudinal PDSR
PSSR-C: The circumferential PSSR
PDSR-C: The circumferential PDSR
CMR: Cardiac magnetic resonance
ICA: Invasive coronary angiography
bSSFP: Balanced steady-state free precession
LVM: LV mass
LVEDV: LV end-diastolic volume
LVESV: LV end-systolic volume
LVSV: LV stroke volume
LVEF: LV ejection fraction
ICC: Intra-class correlation coefficient
GRPS: Global radial peak strain
GCPS: Global circumferential peak strain
GLPS: Global longitudinal peak strain
